# Protocol for investigating the association between *Helicobacter pylori* exposure, antigen-specific serological responses, and resistant versus non-resistant hypertension in a South African cohort

**DOI:** 10.1371/journal.pone.0362215

**Published:** 2026-11-06

**Authors:** Mathilda C. Mennen, Michael Z. Zulu, Koketso C. Mabatha, Richard Baguma, Sango Skelem, Nandipha N. Mbundwini, Olukayode O. Aremu, Marguerite Adriaanse, Daphne van Dijk, Preyanka Pillay, Sam Africa, Erika Jones, Mashiko Setshedi, Adrian Brink, Wendy A. Burgers, Ntobeko A. B. Ntusi

**Affiliations:** 1 Division of Cardiology, Department of Medicine, University of Cape Town, Cape Town, South Africa; 2 Department of Virology, School of Pathology, Faculty of Health Sciences, University of the Witwatersrand, Johannesburg, South Africa; 3 Institute of Infectious Disease and Molecular Medicine, Division of Medical Virology, University of Cape Town, Cape Town, South Africa; 4 Cape Heart Institute, University of Cape Town, Cape Town, South Africa; 5 Institute for Life Science and Technology, Hanze University of Applied Sciences, Groningen, Netherlands; 6 Wits Donald Gordon Medical Centre, Department of Internal Medicine, Faculty of Health Sciences, University of the Witwatersrand, Johannesburg, South Africa; 7 Division of Medical Microbiology, University of Cape Town, Cape Town, South Africa; 8 Division of Nephrology and Hypertension, Department of Medicine, University of Cape Town, Cape Town, South Africa; 9 Kidney and Hypertension Research Unit, University of Cape Town, Cape Town, South Africa; 10 Division of Medical Gastroenterology, Department of Medicine, University of Cape Town and Groote Schuur Hospital, Cape Town, South Africa; 11 South African Medical Research Council, Cape Town, South Africa; Marshall University, UNITED STATES OF AMERICA

## Abstract

**Background:**

Hypertension is a leading contributor to cardiovascular morbidity and mortality, with poor control in low- and middle-income countries. *Helicobacter pylori (H. pylori*) has been associated with hypertension through inflammatory, endothelial, metabolic and immune pathways, but data from sub-Saharan Africa, particularly in resistant hypertension, are limited.

**Methods:**

This cross-sectional observational study will enrol 200 adults aged ≥18 at a tertiary academic hospital in Cape Town, South Africa: 80 with resistant hypertension, 80 with non-resistant hypertension and 40 with normotension. Participants will undergo standardised clinical assessment, blood pressure measurement, and collection of blood, urine and saliva. *H. pylori* exposure will be assessed using the Pylori DuoTect® point-of-care assay, which detects antibodies to CagA and FliD. Laboratory analyses will include lipid profile, inflammatory and nitric oxide-related markers, homocysteine, insulin indices, complete blood count, ghrelin, multiplex cytokine profiling and flow cytometric immunophenotyping. PAXgene® blood RNA samples will be retained for future exploratory analyses. Saliva will undergo exploratory *H. pylori* antigen testing using CerTest® kits, which are validated for stool rather than saliva. Saliva analysis will be exploratory, and results will not define the primary *H. pylori* exposure variable. Remaining saliva will be kept for potential future oral microbiome analyses not defined in this protocol. Multivariable logistic regression will evaluate associations between *H. pylori* exposure and hypertension phenotype, adjusting for demographic, lifestyle, and clinical covariates.

**Discussion:**

To our knowledge, this will be the first African study to integrate point-of-care *H. pylori* serology with detailed clinical, inflammatory, metabolic, and immunological profiling across resistant hypertension, non-resistant hypertension and normotension. Findings may clarify biological pathways linking *H. pylori* exposure to blood pressure dysregulation and inform future mechanistic and interventional studies.

## Introduction

### Hypertension and chronic infections

Hypertension underlies many prevalent cardiovascular diseases worldwide and remains a global health priority, affecting over 32% of women and 34% of men, and contributing to more than 8.5 million deaths annually [1,2]. Between 1990 and 2019, the number of adults living with hypertension doubled to 1.3 billion, with the steepest growth occurring in low- and middle-income countries (LMICs) [1]. In 2019, 82% of all people with hypertension in the world, which amounts to over 1 billion people, lived in LMICs [1]. Fewer than half of people receiving treatment for hypertension, globally, achieve blood pressure control, with global control rates at only 23% in women and 18% in men by 2019 [1]. In sub-Saharan Africa, control rates remained below 13%, showing little improvement since 1990, and a stark contrast to control rates of up to 60% in high-income countries [1].

Poorly controlled or treatment-resistant hypertension is associated with a substantially greater burden of target-organ damage and adverse cardiovascular and renal outcomes, including myocardial infarction, stroke, heart failure, chronic kidney disease and premature mortality [3,4]. Resistant hypertension represents an important high-risk hypertension phenotype and is generally defined as blood pressure remaining above target despite treatment with three antihypertensive agents of different classes, including a diuretic, or requiring four or more agents to achieve control [4]. Its pathophysiology is multifactorial, with recognised contributors including sodium and fluid retention, renin-angiotensin-aldosterone system activation, sympathetic nervous system overactivity, endothelial dysfunction, arterial stiffness, obesity, chronic kidney disease, obstructive sleep apnoea and genetic risk factors [5–7]. Increasing evidence suggests that persistent inflammatory stimuli may contribute to blood pressure dysregulation and treatment-resistant hypertension, providing a rationale for investigating chronic infections in this population. Infectious agents associated with chronic and latent infection, including *Mycobacterium tuberculosis (Mtb)*, *Plasmodium falciparum*, human immunodeficiency virus (HIV), cytomegalovirus (CMV), herpes simplex virus (HSV) and *Helicobacter pylori (H. pylori*), may increase the risk of hypertension and cardiovascular disease [8–11]. Chronic infections can induce persistent low-grade chronic inflammation, a key pathogenic driver of endothelial injury, vascular remodelling, oxidative stress, and altered metabolic and immune pathways involved in blood pressure regulation [12].

### *Helicobacter pylori* and hypertension

*H. pylori* is a highly prevalent chronic gastric pathogen that infects approximately half of the world’s population, with its main reservoir being the human stomach [13]. It is particularly prevalent in many LMICs, infecting 80–90% of individuals in some African countries [13,14]. *H. pylori* is a gram-negative, microaerophilic, flagellated bacterium that colonises the gastric mucosa and is transmitted primarily through the faecal-oral route [13]. However, oral-oral transmission is also possible [13]. Isolation of *H. pylori* in saliva and dental plaque has been reported [13,15,16]. *H. pylori* has adapted to the acidic environment of the human stomach by evolving a range of virulence mechanisms that allow it to withstand highly acidic conditions, colonise the gastric mucus layer, and adhere to epithelial cells [17]. These characteristics enable its effective colonisation of the gastric mucosa, facilitating long-term persistence and chronic gastric inflammation.

Beyond its established roles in gastritis, peptic ulcer disease and gastric cancer, *H. pylori* has been linked to several extra-gastrointestinal conditions, including insulin resistance, metabolic syndrome, vascular inflammation, and cardiovascular disease. Multiple observational studies across Asia, the Middle East and Africa have reported positive associations between *H. pylori* infection and hypertension [18–22]. Similarly, meta-analyses have noted an increased risk of hypertension among individuals with *H. pylori* infection, with pooled odds ratios ranging from 1.3–1.4 [23,24]. A small interventional study conducted in Italy reported reductions in blood pressure following *H. pylori* eradication, offering preliminary support for causality, although its sample size and follow-up were limited [25]. Contrary to the growing body of evidence suggesting a positive association, there are a few studies that reported no association between *H. pylori* infection and hypertension [26,27]. The lack of consensus may be due to strain-specific pathogenicity, with virulence-associated strains such as CagA (cytotoxin-associated gene-A)-positive strains being more strongly linked to cardiovascular outcomes [28]. Duration of infection may also influence risk [29].

### Mechanistic hypotheses

Several interrelated biological pathways provide plausible mechanisms by which *H. pylori* may contribute to hypertension. Virulence-associated strains, particularly CagA-positive strains, may trigger local and systemic inflammation, endothelial dysfunction, and immune cross-reactivity. Anti-CagA antibodies have been shown to cross-react with vascular wall antigens, suggesting a possible autoimmune mechanism [30]. FliD (flagellar hook-associated protein 2) is an essential component of the *H. pylori* flagellar apparatus and is required for functional flagella, motility and effective colonisation of the gastric mucosa [31]. FliD is highly immunogenic and has demonstrated high sensitivity and specificity as a serological marker of *H. pylori* infection [32]. The Pylori DuoTect® assay used in this study detects antibodies to the CagA and FliD antigens, with FliD serving as a marker of *H. pylori* exposure and CagA providing complimentary information regarding exposure to an established virulence-associated antigen. Although, to our knowledge, there is no direct evidence linking FliD itself to cardiovascular disease, its role in motility and colonisation provides a biologically plausible link to persistent bacterial exposure and sustained antigenic stimulation [31]. This is relevant to hypertension, in which chronic inflammatory and oxidative pathways contribute to endothelial dysfunction, vascular remodelling and impaired nitric oxide bioavailability [33,34]. Prospective data from the REGARDS study have linked circulating inflammatory cytokines to incident hypertension, while the CRIC study demonstrated associations between inflammatory biomarkers and apparent treatment-resistant hypertension [35,36]. Studies conducted in diverse population groups have reported associations between *H. pylori* infection and elevated total cholesterol, low-density lipoprotein (LDL) cholesterol, and inflammatory markers, each of which contributes to increased peripheral vascular resistance and heightened hypertension risk [12,37,38]. Chronic inflammatory activation is associated with increased leukocyte counts, homocysteine, fibrinogen, CRP, IL-6 and TNF- α, promoting endothelial dysfunction and altered vascular tone [39]. Inflammation-associated oxidative stress may further reduce nitric oxide (NO) bioavailability, affecting NO functions in vasodilation and vascular homeostasis, including inhibition of platelet aggregation and suppression of smooth muscle cell proliferation [40].

Ghrelin, a hormone predominantly secreted by the stomach that is gastroprotective and plays key roles in gastric mucosal defence, appetite regulation and weight regulation may be disrupted by *H. pylori* infection [41]. Altered ghrelin synthesis can impair insulin sensitivity and glucose uptake, potentially contributing to insulin resistance and metabolic syndrome, both recognised drivers of hypertension [41]. A high dietary salt intake may also promote *H. pylori* colonisation and exacerbate gastric mucosal injury, further amplifying hypertension risk [42].

### Study rationale

Previous observational studies and meta-analyses have reported associations between *H. pylori* infection and hypertension; however, findings have been inconsistent, and most studies have not assessed resistant hypertension specifically. There is a critical paucity of data from sub-Saharan Africa regarding the pathogenic relationship between *H. pylori* exposure and hypertension, particularly resistant hypertension. To date, no African studies have assessed serological markers of exposure to *H. pylori* virulence-associated antigens, including CagA (cytotoxin-associated gene A), or FliD (flagellar hook-associated protein 2) in individuals with resistant hypertension using validated point-of-care assays, or integrated analyses of inflammatory pathways and immunophenotyping in the context of *H. pylori* exposure. Given the high regional burden of *H. pylori* infection and both resistant and non-resistant hypertension, addressing this evidence gap is of substantial public health importance. This study will examine associations between *H. pylori* exposure and hypertension across resistant hypertensive, non-resistant hypertensive and normotensive adults, while exploring the inflammatory, immunological, metabolic and microbial pathways that may underlie these associations.

Accordingly, this study aims to (1) investigate the association between *H. pylori* exposure (including CagA and FliD seropositivity) and both resistant and non-resistant hypertension in a South African cohort; (2) explore biological mechanisms linking exposure to blood pressure dysregulation; and (3) characterise clinical, biochemical, microbial, and immunological features in exposed versus unexposed individuals.

## Materials and methods

### Study objectives

The primary objective of this study is to explore associations between H. *pylori* exposure, specifically serological evidence of exposure to CagA or FliD antigens, and hypertension phenotypes, including resistant hypertension, non-resistant hypertension and normotension. We hypothesise that prevalence of both CagA and FliD seropositivity will be highest in resistant hypertensive participants compared to non-resistant hypertensive and normotensive participants. The null hypothesis will be rejected if inter-group differences reach statistical significance (p < 0.05). Secondary objectives include characterisation of clinical, biochemical, and immunological differences by *H. pylori* exposure status, and identifying potential mechanistic links between exposure and blood pressure dysregulation.

### Study design and sample size

This cross-sectional observational study will be conducted at Groote Schuur Hospital (GSH), a tertiary/quaternary academic hospital affiliated with the University of Cape Town, South Africa (Fig 1). Adult participants, aged ≥18, will be recruited from the hypertension clinic, general medical wards, and the hospital staff cohort. Participants will be classified into one of three groups: resistant hypertension, non-resistant hypertension, or normotension. Participants in the hypertensive groups will be adults with a pre-existing clinical diagnosis of hypertension who are receiving antihypertensive treatment at the time of recruitment. Resistant hypertension will be defined as elevated blood pressure despite concurrent use of three or more antihypertensive medications from different classes, including a diuretic, or adequate blood pressure control on more than three agents [4]. Non-resistant hypertension will be defined as adequate blood pressure control on fewer than or equal to three antihypertensive agents. Normotension is defined as the absence of hypertension and no use of antihypertensive therapy.

**Fig 1:**
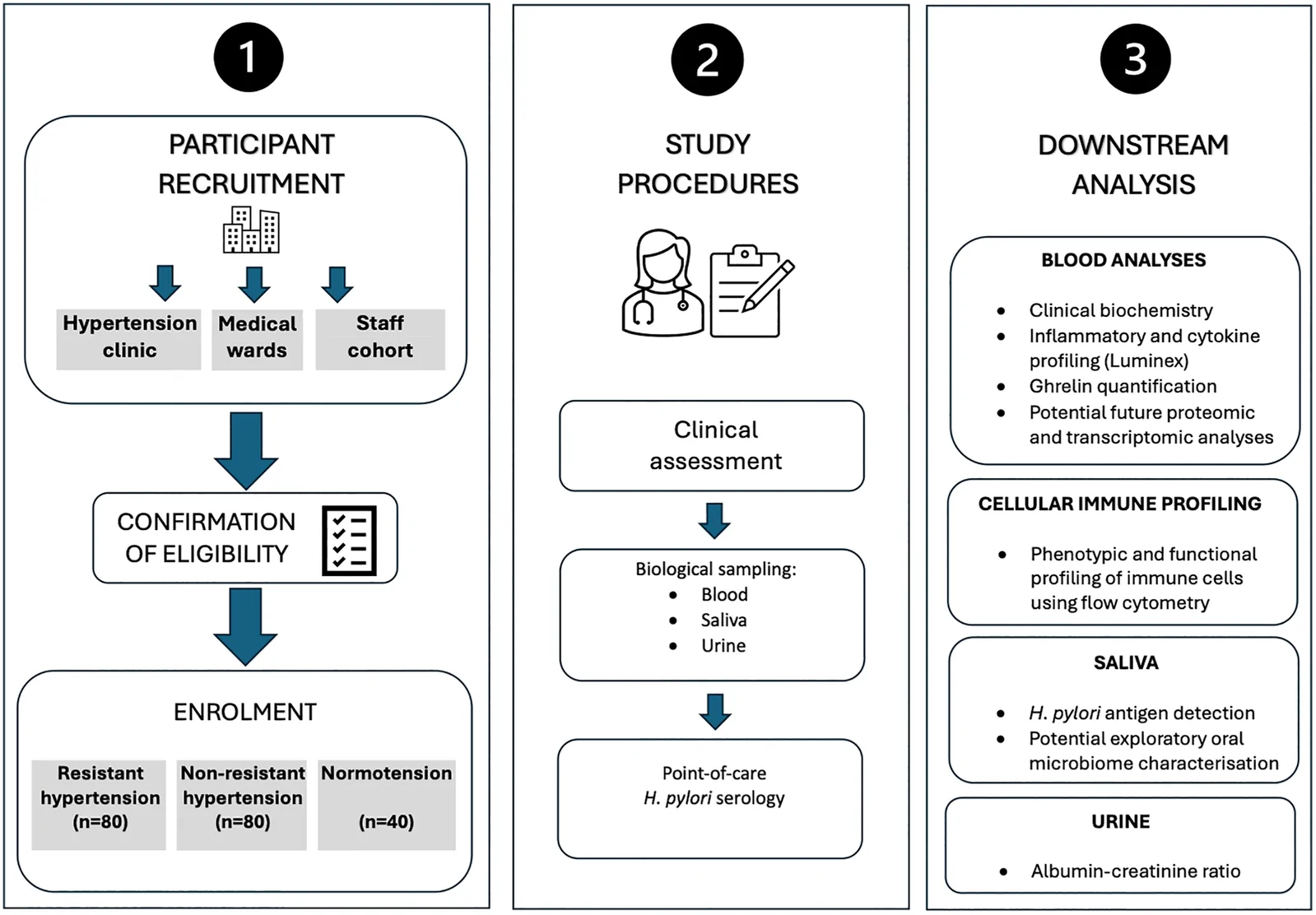
Overview of the study design and protocol workflow. Adult participants will be recruited from a tertiary academic hospital hypertension clinic, general medical wards, and a hospital staff cohort. Following confirmation of eligibility, participants will be enrolled into one of three groups: resistant hypertension (n = 80), non-resistant hypertension (n = 80), or normotensive controls (n = 40). All participants will undergo structured clinical assessment and biological sampling, including blood, saliva, and urine collection. *H. pylori* exposure will be assessed using the Pylori DuoTect® point-of-care assay, which detects antibodies against CagA and FliD. Downstream analyses will include clinical biochemistry, inflammatory and cytokine profiling, ghrelin quantification, potential future proteomic and transcriptomic analyses, immunophenotyping of peripheral blood mononuclear cells, oral *H. pylori* antigen detection, potential exploratory oral microbiome characterisation from saliva, and assessment of albumin-creatinine ratio from urine. The study integrates clinical, microbial, and immunological data to investigate associations between *H. pylori* exposure and resistant versus non-resistant hypertension.

The recruitment target was initially determined pragmatically, based on anticipated recruitment capacity and the exploratory nature of the study. Published meta-analyses have reported modest associations between *H. pylori* infection and hypertension, with pooled odds ratios of approximately 1.3 [23,24]. However, these estimates relate to hypertension in general and do not directly inform the primary comparison between resistant and non-resistant hypertension, for which no reliable prior prevalence or effect-size estimates were available. The planned sample therefore comprises 200 participants: 80 with resistant hypertension, 80 with non-resistant hypertension and 40 normotensive controls. A detectable-effect-size analysis was performed in R version 4.5.2 using a two-sample comparison of proportions, a two-sided α of 0.05 and 80% power. With 80 participants in each hypertensive group, the study is powered to detect an absolute between-group difference in seroprevalence of approximately 21%. The normotensive group will serve as a secondary comparison group for exploratory analyses across hypertension phenotypes.

During recruitment we will match study groups by age, sex, ethnicity and socioeconomic status, and eligibility will be assessed as per the predefined inclusion and exclusion criteria (Table 1). Consecutive eligible patients will be approached during routine clinic visits. This means that all patients attending the relevant clinical services during recruitment periods who meet the eligibility criteria will be approached sequentially for participation, rather than being selectively recruited according to clinical characteristics. Written informed consent will be obtained from all participants in accordance with ethics approval. Consent forms will be witnessed by a study staff member and completed in duplicate, with one signed copy provided to the participant.

**Table 1:** Inclusion and exclusion criteria for study participants.

Inclusion criteria	Exclusion criteria
• Adults ≥18 years • Able to provide written informed consent • Hypertensive and normotensive individuals • Resistant hypertension subgroup prioritised for enrolment	• Inability to consent • Pregnancy • Recent antibiotic use (<4 weeks) • Acute infection • Terminal illness • Pseudo-resistant hypertension • Extensive target organ damage

### Study procedures

Following informed consent, participants will undergo a structured clinical assessment using a case report form approved by the Human Research Ethics Committee. Data collected will include demographics (age, sex, ethnicity), anthropometrics (height, weight), lifestyle factors (smoking, alcohol use, dietary salt intake), comorbidities (hypertension, diabetes, dyslipidaemia, respiratory disease, GI conditions, HIV status), medication history (antihypertensives, statins, proton-pump inhibitors, antibiotics, antiretrovirals, non-steroidal anti-inflammatory drugs and steroids), past medical and surgical history, family history of cardiovascular disease, and vital signs (blood pressure and heart rate). Information will be collected through participant interviews conducted by trained nurses and doctors fluent in English, Afrikaans and isiXhosa, followed by review of their medical records to verify clinical information. Blood pressure will be measured using calibrated automated devices after 5 minutes of seated rest.

### Biological sampling

Consumables will be purchased at the start of the study to avoid batch variability. Samples will be collected in the morning between 09:00 and 11:00 by trained research nurses and doctors under non-fasting conditions. Venous blood will be collected for serum, plasma, peripheral blood mononuclear cells (PBMC) isolation, and RNA extraction. Serum will be collected in a 5 ml VACUTAINER® SST tube; plasma in a 4 ml K2EDTA VACUETTE® tube; PBMCs in two 9 ml Sodium Heparin VACUETTE® tubes; and whole blood in a PAXgene® Blood RNA tube.

Point-of-care *H. pylori* serology will be performed using the Pylori DuoTect® rapid immunoassay to detect antibodies against CagA and FliD antigens. Capillary blood (40 μL) will be collected, applied to the sample port, buffer added, and results interpreted after 20 minutes. The assay contains an intrinsic control line to verify test validity. A positive test will display dark lines alongside the control as well as the CagA and/or FliD labels. A negative test will display a dark line alongside the control label only, and an invalid test will display no control line.

Saliva will be collected using the OMNIgene® ORAL kit (DNA Genotek) which stabilises nucleic acids at ambient temperatures without the need for immediate freezing or cold-chain transport. The kit comes with a collection tube pre-filled with stabilising liquid, a funnel lid, and a cap. Saliva is provided by spitting into the funnel until the liquid volume reaches the marked fill line. Once the funnel is full, the participant closes the lid firmly, which releases the stabilising solution into the saliva. The tube is then secured with a cap and shaken for 5 seconds to mix thoroughly.

Lastly, urine will be collected in sterile containers and secured for transport. After collection, all samples will be processed according to standard operating procedures to ensure sample integrity and analytical reproducibility, and stored on site in −80°C freezers until analysis, as per institutional and regulatory requirements.

### Laboratory experiments

All laboratory analyses will be conducted at accredited University of Cape Town facilities, including the Division of Medical Microbiology, Division of Medical Virology and the Cape Heart Institute.

Samples will be stored at –80°C or in liquid nitrogen until batch analysis. Biomarkers will include lipid profile, high-sensitivity CRP, fibrinogen, homocysteine, insulin indices, complete blood count with differential, and nitric oxide-related metabolites. Serum lipid measurements will include total cholesterol, LDL cholesterol, HDL cholesterol and triglycerides. Plasma samples will be used to measure systemic inflammation and soluble immune mediators. For this we will use the comprehensive Human XL Cytokine Discovery Luminex Panel (R&D Systems, Minneapolis, MN, USA) or similar panel, to measure inflammatory and regulatory cytokines (e.g., IFN-α, IL-17, IL-6, IL-1b, IL-8, TNF- α, IL-10, TGF- b), chemokines (e.g., MCP-1, RANTES, MIP-1α) and other soluble mediators associated with endothelial activation and vascular injury such as vasoactive peptides (endothelial-1), adhesion molecules, soluble VCAM-1, a marker of chronic inflammation in the blood vessels and coagulation and thrombosis marker, von Willebrand factor (vWF). Ghrelin will be measured using validated assays. Whole-blood samples will be collected in PAXgene® Blood RNA tubes for preservation of RNA for future exploratory gene-expression analyses. The specific methodology for this downstream analysis will be defined in a separate protocol and is beyond the scope of the present study.

Peripheral blood mononuclear cells (PBMC) will be isolated from the blood samples using density gradient centrifugation with Ficoll-Paque and cryopreserved in liquid nitrogen until further analyses. PBMC will be thawed, washed and rested, and stained with monoclonal antibodies, with dead cells excluded using a viability dye. Two multicolour flow cytometry panels will be used to characterise innate and adaptive immune compartments. For the detection of monocytes and dendritic cells, we will use an antibody panel optimised for detecting the following markers: CD3, CD20, CD66b, CD56 (exclusion markers), CD16, CD14, HLA-DR, CD11c, CD11b, CD123, CX3CR1, CCR2, CD163, CD64, CD80 and CD86. We will also investigate skewing of adaptive immune cells, namely Th1, Treg, Th17 cells and memory phenotypes, using an adaptive antibody panel consisting of: CD4, CD8, CD19, CCR7, CD45RA, CD27, CD38, HLA-DR, PD1, CD127, CXCR5, CXCR3, CCR6 and CD25. This combination of markers will enable the identification of naïve, memory, and activated T cells and enable delineation of T helper cell subsets. After staining, cells will be washed, fixed and samples acquired on a FACSymphony cytometer using FACSDiva software and analysed using FlowJo (all BD Biosciences). Frequencies and activation states of myeloid and adaptive immune subsets will be compared across participant groups.

All urine and saliva aliquots will be similarly stored at –80°C for batch testing. Albuminuria will be assessed on spot urine for albumin creatinine ratio and stratified as normal (<3 mg/mmol), moderately increased (3–30 mg/mmol), or severely increased (>30 mg/mmol) [43]. It will be included as a marker of renal microvascular injury and hypertension-related target-organ involvement. We will explore whether albumin creatinine ratio differs according to hypertension phenotype and *H. pylori* serological exposure status. Saliva will be tested using CerTest® modified *H. pylori* detection kits (Davies Diagnostics). Because the CerTest® assay is validated for stool rather than saliva, its use in saliva will be treated strictly as an exploratory feasibility analysis. Results will not be used to define the primary *H. pylori* exposure variable or to guide the primary statistical analyses. Remaining saliva will be stored for potential future exploratory oral microbiome analyses not defined in this protocol.

### Statistical analysis

Data analyses will be performed using R (version 4.5.2) and GraphPad Prism version 8.4.4 (GraphPad Software, LLC). Continuous variables will be assessed for normality using the Shapiro–Wilk test and visual inspection. Normally distributed data will be presented as means with standard deviations, and non-normal data as medians with interquartile ranges. Categorical variables will be presented as frequencies and percentages. Group differences will be assessed using appropriate parametric or non-parametric tests for continuous variables and Chi-square or Fisher’s exact tests for categorical variables.

The primary outcome will be resistant hypertension status, with the main comparison being between participants with resistant hypertension and those with non-resistant hypertension. Binary logistic regression will be used to assess the association between *H. pylori* exposure, including CagA and FliD seropositivity, and resistant hypertension. Multivariable models will adjust for potential confounders including age, sex, BMI, smoking status, alcohol use, dietary salt intake, HIV status, relevant comorbidities and medications.

As a secondary analysis, hypertension phenotype will be modelled as a three-level outcome comprising resistant hypertension, non-resistant hypertension and normotension. Multinomial logistic regression will be used with normotension as the reference category to estimate associations of *H. pylori* exposure with resistant hypertension versus normotension and non-resistant hypertension versus normotension. The same covariates will be included in the adjusted models. Effect estimates will be reported as odds ratios with 95% confidence intervals. Statistical significance will be defined as p-value <0.05.

### Study status and timeline

This manuscript describes an ongoing observational study. Participant recruitment began on 11 March 2024 and remains in progress at the time of submission. Recruitment is incomplete, and additional participants will be enrolled to reach the target sample size. Recruitment and data collection are anticipated to be completed by the end of 2026. At submission, none of the primary laboratory experiments or analyses described in the above Methods section have been initiated. All laboratory experiments, statistical evaluations, and results generation will occur following completion of recruitment and biological sample processing.

### Ethics, data management and dissemination

This study was approved by the University of Cape Town Faculty of Health Sciences Human Research Ethics Committee (HREC REF: 795/2023). All study procedures will adhere to the Declaration of Helsinki and the South African Good Clinical Practice 2006 (SA GCP) guidelines. Written informed consent will be obtained from all participants.

Data will be de-identified, stored on password-protected systems, and accessible only to authorised staff. *H. pylori* serology results will be communicated verbally at point-of-care. Participants with a positive salivary *H. pylori* antigen result will be referred to appropriate clinical services for further evaluation and management in accordance with local treatment guidelines. Aggregated findings will be disseminated through departmental presentations and reports. Samples and data will be stored for five years after the final publication arising from the study, and then destroyed. Participants may decline participation or withdraw at any time, in which case their study data and samples will be discarded. Procedural injuries will be covered by UCT’s research insurance policy.

## Discussion

This study will be the first in Africa to integrate clinical phenotyping, point-of-care *H. pylori* serology, and detailed laboratory analysis to investigate microbial contributions to non-resistant and resistant hypertension.

There is a body of literature describing multiple pathways through which *H. pylori* may contribute to hypertension, but findings are inconsistent across populations. The inclusion of rapid serology provides an opportunity to evaluate strain-specific cardiovascular effects in a high-burden setting. We will integrate laboratory analyses to examine pathways through which *H. pylori* exposure may contribute to blood pressure dysregulation.

Limitations include the cross-sectional study design, which precludes causal inference and determining the effect of duration of exposure. Sample size and single-site recruitment may limit generalisability. While point-of-care serological testing using the Pylori DuoTect® assay is pragmatic in resource-limited settings such as ours, sensitivity and specificity may be lower than that of standard clinical diagnostics such as urea breath testing and stool antigen testing. Furthermore, serology alone cannot determine whether infection is currently active, biologically relevant, or temporally related to hypertension. Additionally, performance of the Pylori DuoTect® point-of-care assay has not yet been evaluated in our setting. Convenience sampling from the hospital staff cohort for controls may limit external validity and introduce selection bias, however consecutive sampling of resistant hypertensive cases and non-resistant hypertensive controls from the clinic and the wards will mitigate bias in hypertensive groups. Lastly, the CerTest® assay is validated for stool rather than saliva, and its diagnostic performance in salivary specimens is therefore unknown. Findings from this exploratory salivary analysis will need to be interpreted cautiously and should not be considered evidence of active *H. pylori* infection.

### Expected impact

This study will help clarify the association between exposure to virulence-associated *H. pylori* strains and both non-resistant and resistant hypertension in an African population and may inform targeted microbial screening in high-risk groups. Integrated laboratory analyses will help identify biological pathways underlying this association and guide future mechanistic and intervention studies, including eradication trials.
